# Editorial: Neural computations for brain machine interface applications

**DOI:** 10.3389/fnhum.2023.1334636

**Published:** 2023-11-23

**Authors:** Young Ho Kang, Abed Khorasani, Robert D. Flint, Behraz Farrokhi, Sang Wan Lee

**Affiliations:** ^1^Department of Brain Cognitive Science, Korea Advanced Institute of Science and Technology (KAIST), Daejeon, Republic of Korea; ^2^Kerman Neuroscience Research Center, Kerman Medical University, Kerman, Iran; ^3^Department of Neurology, Northwestern University, Evanston, IL, United States; ^4^Department of Neurology, University of California, San Francisco, San Francisco, CA, United States

**Keywords:** human-AI alignment problem, brain-computer interfaces, BCI, BMI, restorative technologies

Along with a flowering of deep learning, there has been a renaissance of brain-computer interface (BCI) research. One of the most active research areas of the BCI aims attention at a wide range of clinical endeavors, such as studies on prosthetic limb muscle control (Vilela and Hochberg, 2020), neurorehabilitation (Bamdad et al., 2015) or epilepsy (Vidyaratne and Iftekharuddin, 2017; Alkawadri, 2019). A typical clinical application of the BCI aids an individual with central nervous system (CNS) injuries or disabilities, such that it compensates for associated dysfunctions resulting from the impairment. Beyond the scope of clinical purposes, there has also been various research on controlling an external system solely via neural signals (Trejo et al., 2006; Khaliliardali et al., 2015). In this editorial, with insights from carefully selected studies, we explore recent progress in the computational aspects of BCIs. Further, we discuss how such studies can make convergent contributions to benefitting the human-machine alignment.

All five studies on this topic astonishingly broadened our knowledge of computational tools for BCIs. Three of them, in particular, shed light on employing deep learning techniques in various BCIs. Cui et al. contemplated interpretability issues of the BCIs relying upon deep learning. Interpretability is a desirable trait for BCIs; as an interpretable BCI may provide fruitful insight into cognitive and/or neural mechanisms related to kinematics, learning and memory in particular. Deep learning, whilst often boosting decoding performance of the BCI, can be difficult to interpret. This study thus evaluated various interpretation schemes for deep BCI and demonstrated the best practice of utilizing such techniques. Also, Sun et al. introduced the EEG-completion-informer (EC-informer). The authors investigated how the number of EEG acquisition channels can be finely reduced and successfully demonstrated how virtually generated channels can compensate for the information gap. Hence, this approach eliminates extensive computing costs while preserving key factors with minimal loss—exhibiting robust applicability. Yang et al. presented a novel patient-specific approach to predict epileptic seizures based on multimodal neural data and an adversarial model. The domain-adversarial training with the multimodal data enables the model to extract invariant features of individuals and improves the model's stability, which can be otherwise reduced by subject variability. Thereby, this work successfully demonstrated how the BCI can be generalized across the subjects particularly in the clinical domain.

Meng et al. rigorously examined the effect of various gaze fixation positions and covert attention on BCI performance. The work revealed that subjects' performance on the given BCI task was not affected by the position of gaze fixation and covert attention, suggesting, at least for the motor imagery BCIs, the system can be gaze-independent. As a result, the authors provided precise instructions on the extent to which the end-users of BCIs can behave freely. A precise user guide relaxes some burdens on users, granting them a better BCI experience. Unlike the first four non-invasive BCI studies, Wan et al. demonstrated the effects of non-stationary neural signals on an intracortical-BCI (iBCI) decoding performance. Considering recording degradation and neuronal property variation, the study served as a reference for a model and its training scheme in chronic iBCI. The study gave a decent guideline for developing the BCI system with non-stationary neural signals as its input, which is commonly encountered.

Despite a large volume of contributions to advancement of the BCI decoders, in this topic improving interpretability (Cui et al.), generalizability (Yang et al.) and robustness (Sun et al.; Meng et al.; Wan et al.) of the decoder, most BCIs potentially suffer from the human-machine value alignment issues, namely sharing human values with the machine. The alignment theory views both a human and a machine as learning systems, such that two learners actively learn to accomplish a given task through a sequence of interactions within a closed loop. One study (Müller et al., 2017) established a mathematical model for the coadaptation to formulate and solve an asymmetric nature of a communication paradigm of the BCI. They suggested the error rate of human-to-machine communication can minimized by an interactive joint adaptation process via noise-free machine-to-human feedback. Not to mention the significance of the machine's decoding performance to which a large volume of studies have devoted (Craik et al., 2019; Glaser et al., 2020), how crucial it is for users to adequately explore and exploit the BCI has been constantly emphasized as the key factor of a successful brain-actuated system (Perdikis and Millán, 2020).

As a potential solution for the human-machine value alignment problem, deploying a cognitive model in the loop can minimize the burden on two learners. A cognitive model can be defined as a computational model of cognitive processes of interest built based on neural and/or behavioral data. The model's variables reflect various aspects of the cognitive function under investigation from which an inference on an individual's cognitive status can be made, at least within the given context. One good example is the temporal difference (TD) model, initially inspired by animal reward-seeking behaviors (Sutton and Barto, 1987; Sutton, 1988). There has been an attempt to leverage critical variables of the computational model of human reinforcement learning for predicting user intention, showing that the decoder's performance with the given cognitive model can be significantly improved (Kim and Lee, 2018). This model can also be supplemented with a model of human intuition on physics, called an intuitive physics engine (IPE). The IPE makes physical inferences based on human-like physical understanding (Smith et al., 2019). In the context of the BCI system serving a user as a physical aid, for instance, the IPE would generate several possible human-like inferences based on simulations of committing human-like actions. Further, decoded signals and the inferences can be combined to align the machine with the human's demanding objective more precisely. By harnessing the cognitive model, machines in BCIs would become more human-like. As a result, we anticipate the cognitive models to aid machines in understanding intentions with an exponential increase in precision, thereby easing the learning burden of both the machine and the user in the loop.

## Author contributions

YK: Writing—original draft, Writing—review & editing. AK: Writing—original draft, Writing—review & editing. RF: Writing—review & editing. BF: Writing—review & editing. SL: Writing—review & editing.
